# Humidity Effects on Domain Structure and Polarization Switching of Pb(Zn_1/3_Nb_2/3_)O_3_-x%PbTiO_3_ (PZN-x%PT) Single Crystals

**DOI:** 10.3390/ma14092447

**Published:** 2021-05-09

**Authors:** Hongli Wang, Kaiyang Zeng

**Affiliations:** 1 The Key Lab of Guangdong for Modern Surface Engineering Technology, National Engineering Laboratory for Modern Materials Surface Engineering Technology, Institute of New Materials, Guangdong Academy of Sciences, Guangzhou 510650, China; wanghongli@gdinm.com; 2 Guangdong Provincial Key Laboratory of Advanced Energy Storage Materials, School of Materials Science and Engineering, South China University of Technology, Guangzhou 510640, China; 3 Department of Mechanical Engineering, National University of Singapore, 9 Engineering Drive 1, Singapore 117576, Singapore

**Keywords:** relative humidity, PZN-x%PT, domain structure, polarization switching, SPM

## Abstract

The effect of relative humidity on the domain structure imaging and polarization switching process of Pb(Zn_1/3_Nb_2/3_)O_3_-x%PbTiO_3_ (PZN-x%PT) ferroelectric single crystals has been investigated by means of the piezoresponse force microscopy (PFM) and piezoresponse force spectroscopy (PFS) techniques. It was found that the PFM amplitude increases with the relative humidity, and that the ferroelectric hysteresis loops at different relative humidity levels show that the coercive bias decreases with the increase in relative humidity. These observed phenomena are attributed to the existence of the water layer between the probe tip and the sample surface in a humid atmosphere, which could affect the effect of the electric field distribution and screening properties at the ferroelectric sample surface. These results provide a better understanding of the water adsorption phenomena at the nanoscale in regard to the fundamental understanding of ferroelectrics’ properties.

## 1. Introduction

The superior piezoelectric and ferroelectric properties of ferroelectric materials have made them promising candidates for non-volatile random access memories (NVRAMs), microelectromechanical systems (MEMS), high-performance transducers, sensors, and actuators [1,2,3]. These applications are closely related to their domain configurations and polarization states, motivating extensive investigations on the domain structures and polarization switching characteristics of ferroelectric materials [2,4,5]. With the rapid development of the scanning probe microscopy (SPM) technique, it has already been widely used for investigating ferroelectric domains on the micro- and nano-scale [5,6,7]. In particular, piezoresponse force microscopy (PFM) and piezoresponse force spectroscopy (PFS) measurements are commonly used for the direct characterization of the local ferroelectric domain structures and polarization switching behaviors, respectively [8,9,10]. Among these studies, it was noted that the properties of ferroelectric materials could be extremely sensitive to the ambient humidity [11,12,13,14,15].

In the last two decades, thanks to advances in humidity sensor technology [16,17,18], humidity’s effects on ferroelectric materials have attracted numerous research interests and have been studied by theoretical, experimental, and numerical simulation methods [11,19,20,21]. It is generally believed that the formation of a water meniscus between the tip and the sample surface plays a crucial role in the SPM characterization of ferroelectric materials in ambient humid atmospheres [22,23,24,25]. However, there is no consensus on the underlying mechanism by which the water layer affects the property characterization of ferroelectric materials by SPM. Some researchers have ascribed this phenomenon to the existence of the screening charges through ionized species from the water layer present under ambient conditions [21,26,27,28]. Some other researchers have attributed it to the electrocapillary phenomena at the tip–surface junction, and their interaction with bias-induced materials responses [22]. In addition, the observed phenomena were believed to be caused by the redistribution of the switching electric field due to the existence of the conductive adsorption surface layer [29,30]. Since the study of the water adsorption phenomena at the nanoscale is significant for advancing the fundamental understanding of ferroelectric properties, it is essential to clarify the effect of relative humidity on ferroelectrics.

In this work, two SPM techniques, including PFM and PFS, were used to characterize the domain structure and ferroelectric hysteresis loops of PZN-x%PT single crystals under various relative humidities. The purpose of this work was to study the effect of relative humidity on the domain structure imaging and polarization switching properties of PZN-x%PT single crystals, and to explore the possible mechanisms involved therein.

## 2. Materials and Methods

PZN-x%PT single crystals (supplied by Microfine Materials Technology Pte. Ltd., Singapore) grown by an improved flux method were used in this work [31]. After being oriented into (100)^L^(010)^W^(001)^T^ orientations, the samples were cut into dimensions of 4 mm (L) × 4 mm (W) × 0.5 mm (T). Then, the samples were polished to a mirror finish.

A commercial SPM instrument (MFP-3D, Asylum Research, Oxford Instruments, Santa Barbara, CA, USA) was used to carry out the piezoresponse force microscopy (PFM) and piezoresponse force spectroscopy (PFS) measurements for the acquisition of the domain structure and the ferroelectric hysteresis loops, respectively. The PFM and PFS measurements were performed using conductive PtIr-coated silicon tips (l ≈ 150 μm, f ~ 160 kHz, k ≈ 7.4) (PPP-NCSTPt, Nannosensors, Switzerland). Before each measurement, the probe was calibrated to guarantee data reliability. All the SPM measurements were conducted at room temperature (25 °C), which was much lower than the Curie temperature of PZN-x%PT (160 °C) [32]. In the PFS measurements, three loops were obtained at each point to ensure repeatability. The negative (V_N_) and positive (V_P_) switching bias could be extracted from the hysteresis loops, and the coercive bias and imprint bias could be calculated by V_c_ = (V_P_ − V_N_)/2 and V_i_ = (V_N_ + V_P_)/2, respectively [33]. The relative humidity control was achieved through a closed electrical cell (Figures S1 and S2 in the Supplementary Materials). More detailed information can be found in the Supplementary Materials.

## 3. Results and Discussion

**Figure 1:**
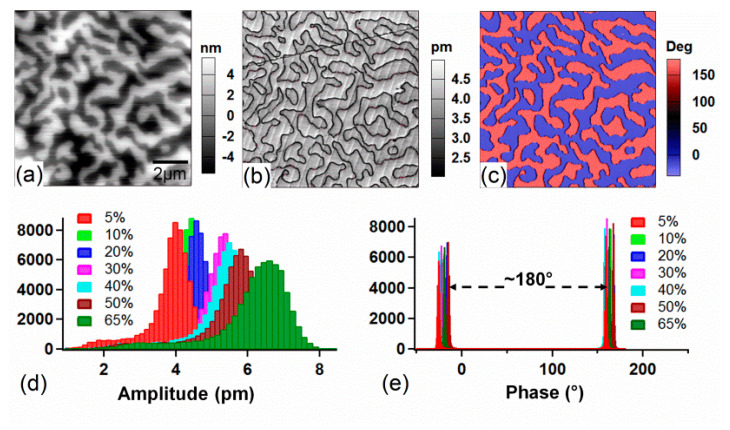
(**a**) Topography, (**b**) PFM amplitude, and (**c**) PFM phase images of the PZN−9%PT single crystal. (**d**,**e**) Histogram distribution of PFM amplitude and phase under various relative humidity levels from 5% to 65%.

To explore the effects of relative humidity on the ferroelectric domain structure imaging, the topography, PFM amplitude, and phase images of the PZN-9%PT single crystal were measured with a scan area of 10 × 10 μm^2^ (Figure 1). It can be seen that there were two types of contrast, and that they had an approximately 180° phase angle difference, which represents two opposite polarity directions, i.e., one was upward and the other was downward [1,34]. Figure 1d,e shows the PFM amplitude and phase distribution with relative humidity at 5%, 10%, 20%, 30%, 40%, 50%, and 65%. It clearly demonstrates that the PFM amplitude increased with the relative humidity, while the phase angle difference remained around 180°, without any significant change with relative humidity. Previous studies have reported that the ambient humidity could affect SPM imaging because a water layer could be formed between the SPM probe tip and the sample surface due to the condensation of water molecules in the surrounding environment [12,21,29]. Therefore, below, we will explore how this water layer affects the PFM amplitude of the ferroelectric domains.

**Figure 2:**
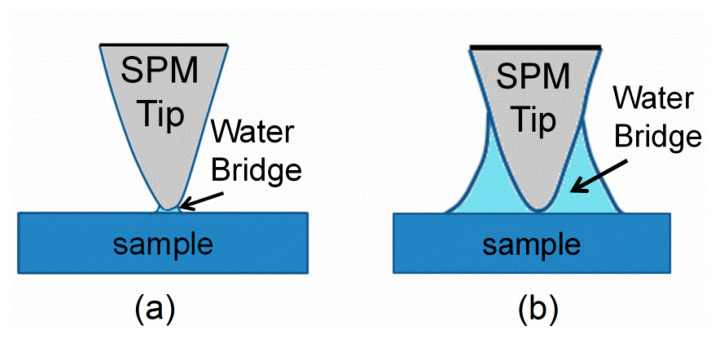
Schematics of the water layer between the tip and the sample surface: (**a**) low relative humidity and (**b**) high relative humidity.

Figure 2 shows the schematics of the water layer between the tip and the sample surface. When the relative humidity was low, the effective contact area of the water layer was relatively small, and the effective contact area increased with relative humidity. This can be validated by the domain growth studies in different relative humidities, which report that the domain size increases with the relative humidity [30,35]. The difference of the effective contact area can affect the electric field strength and distribution between the probe tip and the sample surface. Numerical simulation has revealed that the electric field can be weakened by the thin water layer at low humidity, while being enhanced by the thick water layer at high humidity with the same applied bias [28,36]. This may explain why the amplitude increased with increasing relative humidities from one aspect. Besides, the water layer contains charge species and helps with the formation of the screening layer on the surface of ferroelectric materials [27,28]. This screening layer could weaken or neutralize the depolarization field [22]. Therefore, the effective electric field, which is employed to measure the PFM images, increased at high relative humidities. This could be employed to interpret why the amplitude increased with relative humidity from another aspect.

**Figure 3:**
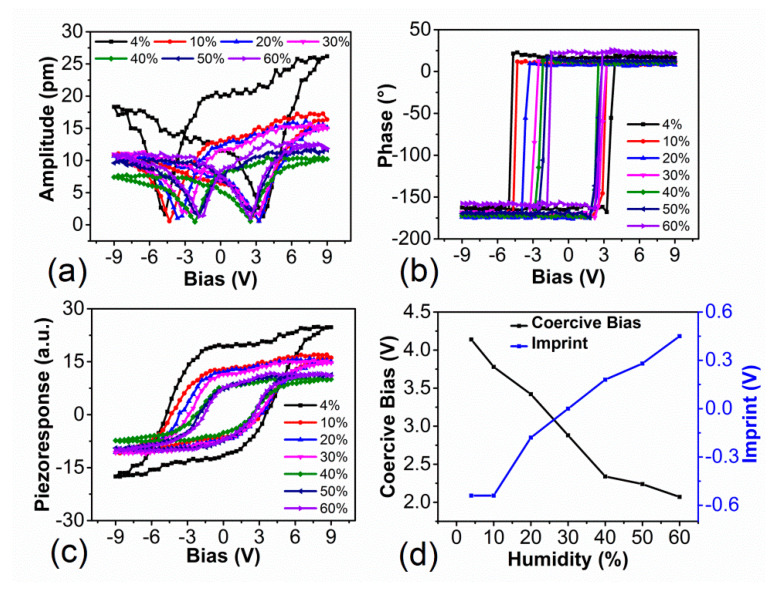
Hysteresis loops of a PZN−9%PT single crystal with relative humidities varying from 4% to 60%: (**a**) amplitude loop, (**b**) phase loop, and (**c**) calculated piezoresponse loop. (**d**) Coercive bias and imprint as a function of the humidity.

Relative humidity affected not only the PFM imaging process, but also influenced the characterization of the polarization switching properties through the adsorption of molecular water and ambient charge species [12,20,21,29,36]. Hence, the effects of relative humidity on the ferroelectric switching behavior of PZN-9%PT single crystal were investigated. Figure 3a–c shows the amplitude, phase, and piezoresponse hysteresis loops of a PZN-9%PT single crystal with the relative humidity in the range from 4% to 60%. The coercive bias and imprint (Figure 3d) were extracted from the mathematical analysis of the loops [37]. It clearly shows that the coercive bias decreased with relative humidity. This phenomenon can be ascribed to the abovementioned water layer formed between the probe tip and the sample surface [27,28]. This water layer can influence the switching behavior in two aspects. One is that this water layer changes the electric field strength and distribution [20,29,36], and the other is that this water layer affects the sample surface screening properties of ferroelectric materials through the adsorption of charge carriers [21,27]. Since the electric field was weakened at a low relative humidity, while enhanced at high relative humidity [28,36], that is why the coercive bias decreased with the relative humidity.

Figure 3d also shows that the imprint increases with the relative humidity. Previous studies have shown that the imprint of ferroelectric materials can be affected by various factors, including the screening layer through surface trapped charges species [38,39,40]. With the increase in the relative humidity, the amount of adsorbed water molecules increased, and more charge species were introduced. These charge species were bound to the ferroelectric material surface and favored one specific polarization direction. In this work, the hysteresis loops were obtained in the domains with downward direction, and the imprint shifted towards the direction that preferred the downward direction. This indicates that the adsorbed water molecules can help to stabilize the original preferential polarization direction, which is consistent with the results of previous studies [11,27].

**Figure 4:**
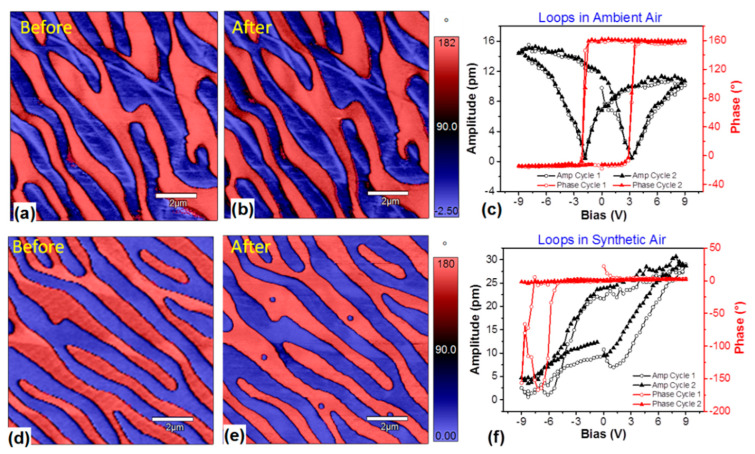
(**a**,**d**) PFM phase images of PZN−9%PT before PFS in the ambient air and the synthetic air, respectively; (**b**,**e**) PFM phase images after PFS in the ambient air and the synthetic air, respectively; and (**c**,**f**) hysteresis loops in the ambient air and the synthetic air, respectively.

To further validate the effect of the water layer on the polarization switching properties of PZN-x%PT single crystals, hysteresis loops in the ambient air (with a humidity of 60–70%) and in synthetic air (21% oxygen, 79% nitrogen, and moisture content < 5 ppm) were obtained (Figure 4). In our experiments, the relative humidity was adjusted by mixing different ratios of synthetic air and ambient air in a closed cell (Figures S1 and S2 in the Supplementary Materials). When the relative humidity was extremely low (<1%), the closed cell was almost entirely filled with synthetic air, which contained only pure nitrogen and oxygen, without water molecules or charge carriers. Figure 4c,f show the hysteresis loops obtained in the ambient air and the synthetic air, respectively. It can be seen that the hysteresis loops obtained in the ambient air demonstrated typical shapes of the ferroelectric materials, i.e., the amplitude loop showed a butterfly shape, and the phase loop presented an approximately 180° difference. In contrast to the hysteresis loops obtained in the ambient air, the hysteresis loops acquired in the synthetic air did not show any obvious polarization switching characteristics; the amplitude loops displayed an irregular rhombus shape, and the phase loops showed a certain degree of oscillations, which are smaller than 180°, suggesting that the polarization switching process was not successfully achieved in the synthetic air atmosphere.

In addition, the PFM phase images before and after the polarization switching processes in different atmospheres are shown in Figure 4a,b,d,e. For the phase images acquired in the synthetic air, the marks of the PFS points are clearly seen in the PFM phase images after the PFS measurements, while no obvious marks can be seen in the PFM phase images obtained after the PFS measurements in the ambient air. This difference can be explained by the presence of humidity. For the hysteresis loops obtained in the ambient air, due to the presence of humidity, which contains free charge carriers, the charges generated during the PFS process can be compensated. For the hysteresis loops obtained in the synthetic air, there was not sufficient humidity to provide free charge carriers to compensate for the tip-generated charges. Therefore, the charges generated during the PFS process accumulated on the surface of the sample. That is why the PFS points are visible in the PFM images obtained in the synthetic air. Considering the phase images and the hysteresis loops obtained in different atmospheres, it is believed that the polarization switching process can be promoted by the charge carriers and moisture in the ambient air. Based on the different results in synthetic air and ambient air, it can be concluded that the charge carriers and moisture in the ambient air can facilitate the polarization switching of PZN-x%PT single crystals, i.e., reduce the coercive bias for polarization switching.

## 4. Conclusions

In this study, the effects of relative humidity on the domain structure imaging and polarization switching properties of PZN-x%PT single crystals were investigated by PFM and PFS techniques. It was found that the PFM amplitude increased with the relative humidity, while the PFM phase had no significant relative humidity dependence. In addition, the ferroelectric hysteresis loops at different relative humidity levels showed that the coercive bias decreased with the relative humidity, while the imprint increased with the relative humidity. The water layer formed between the probe tip and the sample surface due to the condensation of water molecules in the environment was employed to explain the above results. Specifically, the electric field was weakened with the thin water layer at a low relative humidity, while it was enhanced with a thick water layer at a high relative humidity. The study on the polarization switching behaviors in the ambient air and synthetic air revealed that the coercive bias in synthetic air was larger than that in the ambient air, suggesting that the charge species and moisture in the ambient air can facilitate the polarization switching process. These results provided a better understanding of the experimental contribution of the water layer to the domain structure and ferroelectric hysteresis loop measurements of ferroelectric relaxors in SPM studies.

## Data Availability

Data sharing is not applicable to this article.

## Supplementary Materials

The following are available online at https://www.mdpi.com/article/10.3390/ma14092447/s1, Figure S1: Images of the closed electrical cell: (a) the top part; and (b) the bottom part., Figure S2: (a) schematic of humidity control system; and (b) image of the humidity control system used in this study.
