# Supplementary material for: Humidity Effects on Domain Structure and Polarization Switching of Pb(Zn1/3Nb2/3)O3-x%PbTiO3 (PZN-x%PT) Single Crystals
Source: Materials (Basel). 2021 May 9;14(9):2447. doi: 10.3390/ma14092447 (PMC8125925; doi:10.3390/ma14092447)
Supplement: Supplementary file 1 [file materials-14-02447-s001.zip › materials-1206612-supplementary.pdf]

materials

Supplementary Materials

Humidity Effects on Domain Structure and Polarization Switching of $Pb(Zn_{1/3}Nb_{2/3})O_3$-x%$PbTiO_3$ (PZN-x%PT) Single Crystals

Hongli Wang [1,2] and Kaiyang Zeng [3,*]

[1] The Key Lab of Guangdong for Modern Surface Engineering Technology, National Engineering Laboratory for Modern Materials Surface Engineering Technology, Institute of New Materials, Guangdong Academy of Sciences, Guangzhou 510650, China; wanghongli@gdinm.com
[2] Guangdong Provincial Key Laboratory of Advanced Energy Storage Materials, School of Materials Science and Engineering, South China University of Technology, Guangzhou 510640, China
[3] Department of Mechanical Engineering, National University of Singapore, 9 Engineering Drive 1, Singapore 117576, Singapore
* Correspondence: mpezk@nus.edu.sg; Tel: (+65)-6516-6627; Fax: (+65)-6779-1459

In our experiment, a commercial Humidity Sensing Cell (Asylum Research, Oxford Instruments, Santa Barbara, CA, USA) was used to detect the real-time humidity. As shown in Figure S1, there are four holes in edge of the closed cell, 1 and 2 for dry and wet gas in, 3 and 4 are for gas out. The humidity can be controlled by dynamically filling the closed cell with different ratios of wet and dry gases.

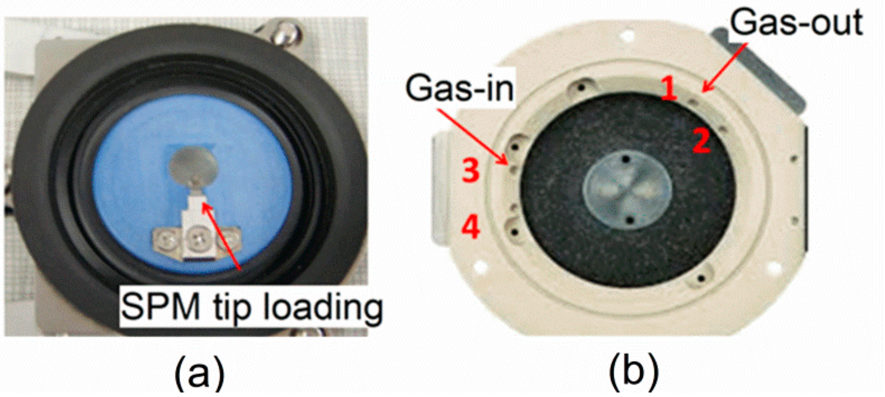

Figure S1. Images of the closed electrical cell: (**a**) the top part; and (**b**) the bottom part.

As shown in Figure S2, the ratio of dry and wet synthetic air can be controlled by flowmeter 1 and flowmeter 2. By adjusting the flow rate, the relative humidity manipulation can be achieved. At the same time, an environmental controlling cell with a humidity sensor is employed to detect the real-time relative humidity during the SPM measurements.

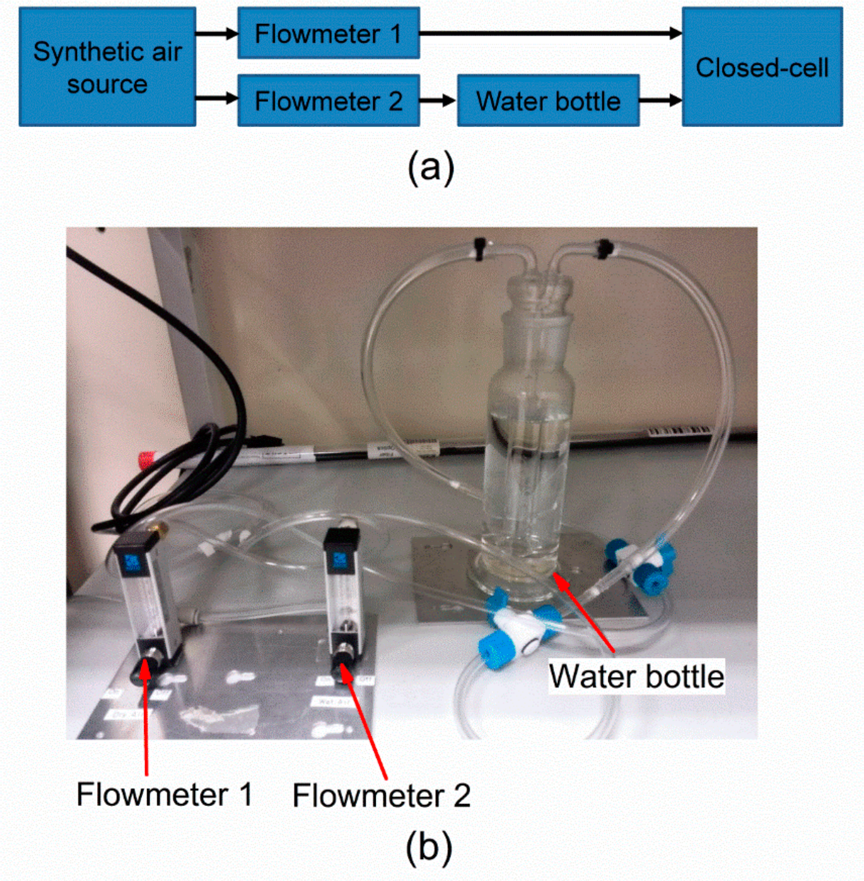

Figure S2. (**a**) schematic of humidity control system; and (**b**) image of the humidity control system used in this study.
